# Respectful maternity care during facility-based child birth and associated factors in public health facilities of Ilu AbaBor zone, Southwest Ethiopia

**DOI:** 10.1186/s12978-022-01368-z

**Published:** 2022-04-22

**Authors:** Worke Sisay Yismaw, Tigist Shayi Teklu, HariPrasath Panduragman

**Affiliations:** Department of Nursing, College of Health Sciences, Mettu University, Mettu, Ethiopia

**Keywords:** Compassion, Respect, Ilu Ababor zone, Childbirth, Pregnant

## Abstract

**Background:**

Current data suggest that far more women around the world are exposed to abuse by health care providers while receiving maternity care. This predisposes them to psychological distress; abstain from accessing health facilities for care and end up with avoidable death and disability.

**Objective:**

To assess the level of respectful care during delivery among mothers giving birth in public health facilities in Ilu AbaBor, Southwest Ethiopia, 2019.

**Materials and methods:**

An institution-based cross-sectional study was conducted among 285 randomly selected mothers in the post-natal ward from 17 health facilities in 2019. Data were collected through interviewer-administered pre-tested questionnaires. The collected data were entered into Epi-data version 3.1 statistical and analyzed by SPSS version 21. Both bivariable and multivariable logistic regression models were employed.

**Results:**

The proportion of respectful care during maternity care in health care institutions of Ilu Ababor zone was 47.3%. In the multivariable logistic regression; age (AOR 0.25, 95% CI 0.08, 0.81), occupation (AOR 4.16: (1.34, 12.9)), planned pregnancy (AOR 0.28: (0.12, 0.67)), mode of delivery (AOR 0.05: (0.01, 0.33)), and receiving care from providers who had a compassionate and respectful care training (AOR 0.13: (0.06, 0.25)) were independent predictors of respectful care.

**Conclusions:**

The proportion of respectful care of the health institution in the Ilu Ababor zone was low compared to the other studies. Variables like; age, occupational status, pregnancy plan, mode of delivery, facing complications during labor and delivery, and taking compassionate and respectful care training were independent predictors of respectful maternity care. This study recommended that the responsible stakeholders should strengthen monitoring and evaluation of the practice and mainstreaming of respectful maternity care, give training for health professionals, and develop guidelines used to monitor, report, and track barriers to the practice of maternity care.

## Plain English Summary

Respect during maternity care advocates that females’ experiences of delivering are a crucial part of service standard: autonomy, dignity, feelings, choices, and preferences must be valued. If a facility is not providing care properly, it affects utilization of maternity services and their satisfaction is also affected by the way they are treated.

Two hundred eighty-one (281) post-natal women participated in this research, giving a 98.5% response rate. Most (34.2%) of mothers were in the age category of 25 to 29 years, attained at least primary education (40.9%) while most (87.2%) of them are married, housewives (32%) in occupation, Oromo (66.9%) in ethnicity with a monthly income of less than 1200 ETB (52.3%) and urban (51.2%) residents.

In conclusion, the proportion of respectful care at the health institution of Ilu Ababor zone is 47.3%, which is low. Age, occupational status, pregnancy plan, mode of delivery, facing complications during labor and delivery, and taking respectful care training were independent predictors of respectful care at the health facilities. This study recommended that the responsible stakeholders should strengthen monitoring and evaluation of the practice and mainstreaming of respectful maternity care, give training for health professionals, and develop guidelines used to monitor, report, and track barriers to the practice of maternity care.

## Introduction

Respect during maternity care (RMC) advocates that females’ experience of delivery is a crucial part of service standard; “autonomy, dignity, feelings, choices, and preferences must be valued” [1, 2].

Current data indicate that many women globally are exposed to miss-treatment by health care providers while getting maternity care. This will predispose them to emotional distress; make them abstain from visiting health sectors for delivery services and end up with avoidable death and disability [3, 4]. The most commonly raised reason for the low number of deliveries by skilled birth attendants is the lack of RMC which is explained by disrespect and abuse done by health workers [5].

Not respecting and abusing mothers in obstetrics hinder them from receiving care from professionals. It may result in; the death of mothers, newborns, and different birth injuries. Despite severe impacts, it's rarely disclosed, especially in developing nations like Ethiopia [4]. Similarly, reviewed literature by the maternal and child health integrated program (MCHIP) in Ethiopia found that perceptions of women on the cleanliness of institutions, availability of materials, and competency of the providers or their behavior were found to be barriers to delivery utilization in facilities [6]. Moreover, Mother’s childbirth experience is the other important factor for future decisions related to willingness to seek care from institutions [7].

If a facility fails to provide care properly, it affects utilization of maternity services, and their satisfaction [8, 9]. There is scarcity of information on the presence of disrespect and abuse in facilities-based childbirth services given in limited-resource countries. However, it is not disclosed by the data recorded by health facilities [10, 11]. The study conducted in Kenya, Tanzania, Ethiopia, and Nigeria showed that the level of RMC was (80%, 72–80%, 22%, and 2%, correspondingly) [9, 12]. This means the level of RMC is low. Since the government is focusing on compassionate and respectful care to increase institutional service delivery, RMC is a current issue in Ethiopia. To our knowledge, there is no published study on respectful maternity care in the Ilu Ababor zone. So, the purpose of this research was to estimate the level of RMC and its predictors in health institutions of Ilu Ababor zone, southwest Ethiopia.

## Methods

### Study setting and period

A facility-based cross-sectional study was conducted in the Ilu Ababor zone from November 1 to 30/2019. It is one of the zones in the Oromia regional state in Ethiopia. It has 1,271,609 total populations of whom 636,986 are men and 634,623 women. Mettu is the capital city of the zone and is 600 km distance from Addis Ababa, the capital city of the country. There are two public hospitals and thirty-nine health centers in the zone.

### Population and sampling

All women who delivered at the 17 health facilities of Ilu Ababor were included and those who were severely ill were excluded from the study during data collection. The sample size was calculated using the single population proportion formula by considering the prevalence of respectful care which was 21.4 in the previous study [13] and a 95% confidence interval which resulted in a final sample size of 285. A systematic random sampling technique was employed. The sample size was proportionally allocated to health facilities on the basis of the client flow reports of previous months before the study was done. We used the lottery method to the first study unit and then every 4th interval to get a required sample.

### Data collection tools and procedures

A structured questionnaire was utilized to collect the necessary data for the research. The questionnaire was adopted from literature and MCHIP [14]_._ The questionnaire has been prepared in English, and then it was translated to Afan-Oromo and Amharic versions by language experts. It was prepared to collect data on socio-demography and other factors. Data were collected by nine trained diploma midwives and supervised by four MSc. holders. Respectful care was measured using 15 items on four components which include; friendly, abusive-free, timely, discrimination-free care (mean value) [14, 15].

### Data processing and analysis

The collected data were entered into Epi-data version 3.1 software and analyzed using 21 versions of SPSS. Frequencies, consistencies, and missed values were checked. Results were compiled and presented using tables, figures, and texts. In addition to this, bivariable logistic regression was carried out to check the association of each of the independent variables with respectful maternity care which is the outcome variable. Thereafter, the multivariable logistic regression was used to identify independent predictors of respectful maternity care. Variables with a P-value of < 0.05 at a 95% confidence interval were declared as statistical significance with the outcome variable.

## Results

### Socio-demographic characteristics of respondents

A total of 281 postnatal women were included in the study making a 98.5% response rate. More than one-third (34.2%) of mothers were in the age category of 25 to 29 years. The majority (37%) of the participants were protestant followers. Regarding educational status, 40.9% of them attended primary education. Furthermore, most (87.2%) of them were married. Two third (66.9%) of the study participants were Oromo and more than half (52.3%) earn a monthly income of less than 1200 (Table 1).

**Table 1:** Socio-demographic characteristics of postnatal women in Ilu Ababor zone public health facilities, Southwest Ethiopia, 2019 (n = 281)

Variable	Category	Frequency	Percent
Age in years	15–19	36	12.8
	20–24	93	33.1
	25–29	96	34.2
	30–34	36	12.8
	35 and above	20	7.1
Religion	Orthodox	74	26.3
	Muslim	103	36.7
	Protestant	104	37
Educational status	Unable to read and write	63	22.4
	Primary education	115	40.9
	Secondary education	65	23.1
	Higher education	38	13.5
Marital status	Married	245	87.2
	Single	22	7.8
	Widowed	6	2.1
	Divorced	8	2.8
Occupation	House wife	90	32.0
	Farmer	79	28.1
	Student	29	10.3
	Government employee	42	14.9
	Merchant	41	14.6
Ethnicity	Oromo	188	66.9
	Amhara	45	16.0
	Tigray	11	3.9
	Gurage	22	7.8
	Others	15	5.3
Monthly income	≤1200	147	52.3
	> 1200	134	47.7
Residence	Urban	144	51.2
	Rural	137	48.8

### Obstetric characteristics of respondents and category of respectful maternity care

**Fig. 1:**
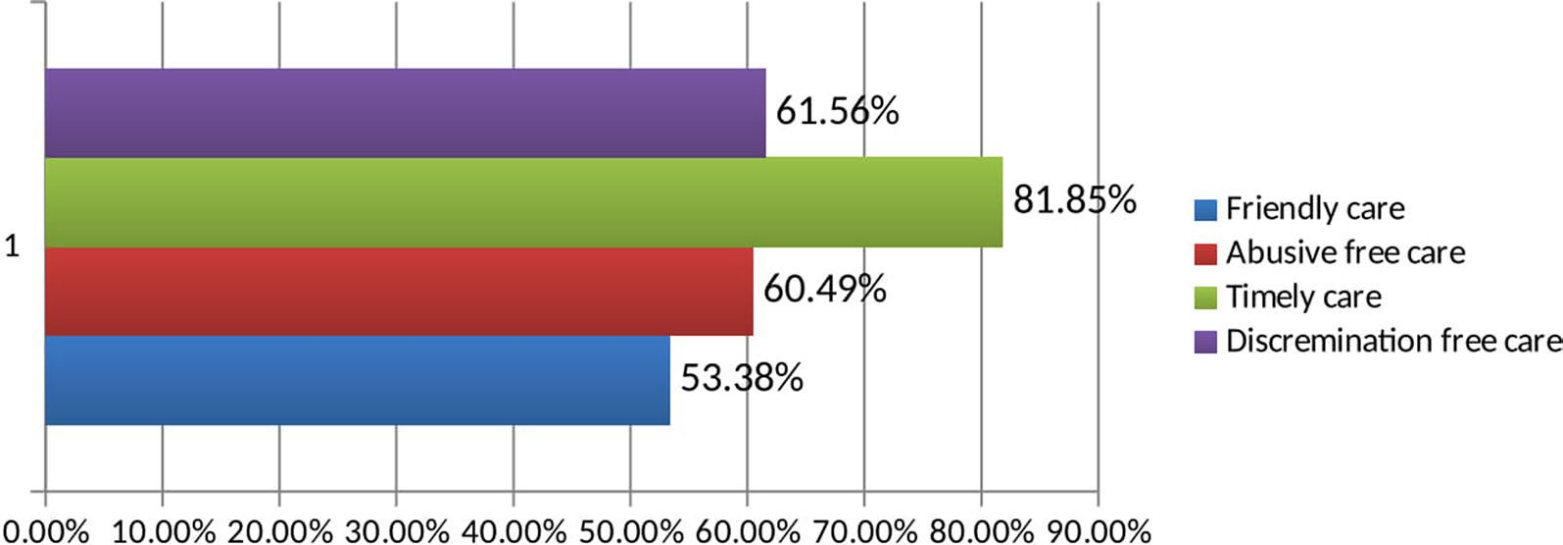
Prevalence of each category of respectful maternity care in Ilu Ababor zone public health facility, Southwest Ethiopia, 2019 (n = 281)

**Fig. 2:**
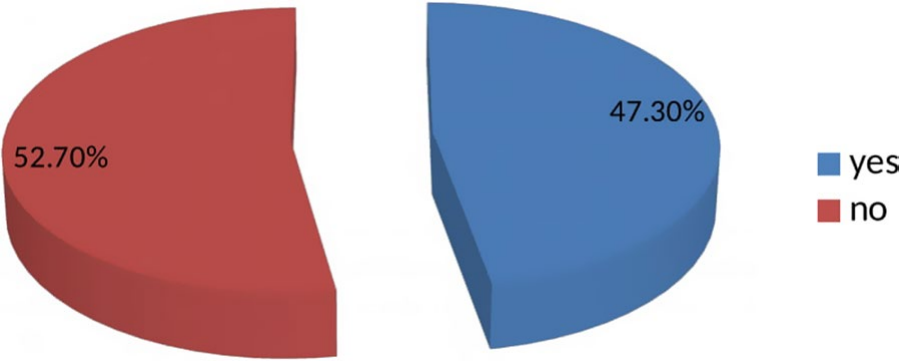
Prevalence of compassionate and respectful maternity care during facility based child birth,in Ilu Ababor zone public health facility, Southwest Ethiopia, 2019 (n = 281)

Half (50.9%) of the respondents had given birth 1–2 times. Almost nine out of ten women (92.9%) had ANC visits. Nearly three-quarters (73%) had planned pregnancy and 207 (73.3%) had spontaneous vaginal delivery performed by 162 female health care providers (Table 2), (Figs. 1, 2).

**Table 2:** Obstetric characteristics of post natal women in Ilu Ababor zone public health facilities, Southwest Ethiopia, 2019 (n = 281)

Variable	Category	Frequency	Percent
Delivery	1–2	143	50.9
	≥ 3	138	49.1
ANC visit	Yes	261	92.9
	No	20	7.1
Pregnancy plan	Yes	205	73
	No	76	27
Current mode of delivery	Spontaneous vaginal delivery	207	73.7
	Cesarean section	56	19.9
	Instrumental delivery	18	6.4
Sex of the provider	Female	162	57.7
	Male	119	42.3
Complication encountered	Yes	58	20.6
	No	223	79.4
No of day spent	≤1	194	69
	2–3	65	23.25
	≥ 4	22	7.75

### Predictors of respect during maternity care

In the bivariable logistic regression analysis; age, educational status, occupation, number of deliveries, antenatal care, pregnancy plan, mode of delivery, compassionate and respectful training, and facing complications were factors associated with respectful maternity care. In the multivariable logistic regression; age, occupation, pregnancy plan, mode of delivery, compassionate and respectful training, and facing complications maintained their association with respectful maternity care. Respondents whose age 15–19 years were 75% less likely to have respectful care (AOR 0.25, 95% CI 0.08, 0.81) compared to over 20 years, students were four times more likely to have respectful maternity care (AOR 4.16, 95% CI 1.34, 12.9) as compared to housewives. Respondents who don't plan their pregnancy were 72% less likely to have respectful care (AOR 0.28, 95% CI 0.12, 0.67) as compared to those who had planned pregnancy. Mothers who had instrumental delivery were 95% less likely to have respectful care (AOR 0.05, 95% CI 0.01, 0.33) as compared to mothers who delivered by spontaneous vaginal delivery. Mothers who received care from providers who had no compassionate and respectful training were 87% less likely to have respectful care (AOR 0.13, 95% CI 0.06, 0.25) as compared to mothers who received care from providers who had training. Women who developed complications in labor were 74% less likely to get respectful maternity care (AOR 0.26, 95% CI 0.10, 0.66) as compared to those with no complications (Table 3).

**Table 3:** Bivariate and multivariable logistic regression analysis of respectfully maternity care among mothers during childbirth, in Ilu Ababor zone public health facilities, southwest Ethiopia, 2019

Variable	RMC		COR (95%CI)	AOR (95%CI)
	Yes	No		
Age				
15–19 years	11 (8.3)	25 (16.9)	1	1
20–24 years	47 (35.3)	46 (31.1)	0.43 (0.19, 0.98)	0.25 (0.08, 0.81)*
25–29 years	44 (31.3)	52 (35.1)	0.52 (0.23, 1.18	0.19 (0.06,0.62)**
30–34 years	18 (13.5)	18 (12.2)	0.44 (0.17, 1.15)	0.20 (0.05, 0.73)*
≥ 35 years	13 (9.8)	7 (4.7)	0.24 (0.07, 0.76)	0.04 (0.01, 0.19)**
Occupational				
House wife	46 (34.6)	44 (29.7)	1	1
Farmer	31 (23.3)	48 (32.4)	1.62 (0.88, 2.99)	9.4 (1.29, 18.39)
Merchant	22 (16.5)	19 (12.8)	0.90 (0.43, 1.89)	0.96 (0.38, 2.40)
Students	12 (9)	17 (11.5)	1.48 (0.64, 3.45)	4.16 (1.34, 12.9)*
Gov’t employ	22 (16.5)	20 (13.5)	0.95 (0.46, 1.98)	1.13 (0.19, 6.82)
Pregnancy plan				
Planned	110 (82.7)	95 (64.2)	0.38 (0.21, 0.66)	0.28 (0.12, 0.67)**
Not planned	23 (17.3)	53 (35.8)	1	1
Mode of delivery				
Spontaneous vaginal delivery	105 (78.9)	102 (68.9)	0.12 (0.03, 0.54)	0.05 (0.01, 0.33)**
Cesarean section	26 (19.5)	30 (20.3)	0.14 (0.03, 0.69)	0.05 (0.01, 0.34)**
Inst. Assisted	2 (1.5)	16 (10.8)	1	1
Compassionate and respectful training				
Taken	87 (65.4)	37 (25)	0.18 (0.11, 0.30)	0.13 (0.06, 0.25)**
Not taken	46 (34.6)	111 (75)	1	1
Facing complications				
Yes	14 (10.5)	44 (29.7)	1	1
No	119 (89.5)	104 (70.3)	0.28 (0.14, 0.54)	0.26 (0.10, 0.66)**

*Significantly associated

**Highly significantly associated

## Discussion

In this study, the proportion of respectful maternity care was found to be 47.3%. This result is higher than study conducted in Pakistan (0.3%) [9] and Nigeria (2%) [16], and it is lower in prevalence than studies conducted in Kenya (80%) [17], Addis Ababa (84%) [13] Tigray (75.6%) [18] Tanzania (81%) [19], Bahirdar (57%) [20]. The difference in the results may be due to variation in methodology, study time, socio-economy of the study groups, health policy, facility, culture, and infrastructure.

Respondents whose ages were between 15 and 19 years were less likely to have respectful care compared to respondents over 20 years of age. This finding was similar to the study findings from Tanzania [19], Kenya [17], and Tigray [18]. The similarity may be due to the same study population and methodology. Moreover, being students were more likely to take respectful maternity care as compared to housewives. This finding is consistent with research conducted in Tigray [18], pakistan [9], and Tanzania [19].

In contrary to other studies, respondents who planned their pregnancy were more likely to get respectful care as compared to those who had not. This is maybe those with planned pregnancies may have a high probability of attaining antenatal follow up mainly in the same facility for delivery service which will, in turn, facilitate their good interaction with health professionals.

Furthermore, respondents who had instrumental delivery were less likely to get respectful care as compared to spontaneous vaginal delivery. This finding is consistent with the study conducted in Nigeria [16]. This is maybe those with assisted delivery will suffer from a lot of stress or pain to the procedure and will have an impact on remembering the real situation at that time.

Mothers who received care from providers who had not trained on compassionate and respectful care were less likely to have respectful care as compared to those who received care from providers who were trained. This finding is consistent with the study conducted in rural Tanzania [21]. This is might be, CRC training which may influence their knowledge, incite and attitude regarding the issue which will have a great impact on the practice of RMC.

Contrary to other studies, respondents who develop complications during labor were less likely to get respectful maternity care as compared to those with no complications. This may imply that the complication is alarming to health professionals with the care as they need special attention which is part of RMC. There is no other study that identified the association between complications during labor and respectful care.

The strength of this study is that we used appropriate statistical tests and get a high response rate. However, the paper is not without limitations. The cross-sectional nature of the study; the study used a cross-sectional study design; hence it is not possible to establish a cause-effect relationship between the variables.

## Conclusions

In this study, the proportion of RMC was found to be low as compared to other previous studies. Variables like; age, occupational status, pregnancy plan, mode of delivery, facing complications in labor, and compassionate and respectful training were predictors of respectful maternal care. This study recommended that the responsible stakeholders should strengthen monitoring and evaluation of the practice and mainstreaming of respectful maternity care, give training for health professionals, and develop guidelines used to monitor, report, and track barriers to the practice of maternity care. It is better to do further research on the same issue in the other parts of the country to come up with more representative results.

## Data Availability

All data and materials which support the evidence are available and given upon reasonable request.

## Abbreviations

ANC: Antenatal care
AOR: Adjusted odds ratio
CI: Confidence interval
CRMC: Compassionate and respectful maternity care
D&A: Disrespect and abuse
HF: Health facility
HSTP: Health sector transformation plan
MCHIP: Maternal and child health integrated program
MMR: Maternal mortality ratio
RMC: Respectful maternity care
SBA: Skilled birth attendant
SPA: Service Provision Assessment
WHO: World health organization

## Declarations

### Patient and public involvement

The public and patients were not involved in preparing a research design, conducting a study, developing tools, and disseminating of research results. But they are involved in the data collection process.
